# Early recurrent midgut volvulus post Ladd’s procedure in a newborn: a case report

**DOI:** 10.1093/jscr/rjaf428

**Published:** 2025-06-18

**Authors:** Nicole Clarke, Joanna Sajdlowska, John Paul Bustamante, Nishith Bhattacharyya

**Affiliations:** Rowan-Virtua School of Osteopathic Medicine 1 Medical Center Drive, Stratford, NJ 08084, United States; General Surgery Department, St. Joseph University Medical Center, 703 Main St, Paterson, NJ 07503, United States; General Surgery Department, St. Joseph University Medical Center, 703 Main St, Paterson, NJ 07503, United States; General Surgery Department, St. Joseph University Medical Center, 703 Main St, Paterson, NJ 07503, United States

**Keywords:** malrotation, Ladd’s procedure, midgut volvulus, adhesions, case report

## Abstract

Ladd’s procedure is the standard surgical intervention for intestinal malrotation. Although generally curative, rare cases of early postoperative complications, including adhesions or recurrent volvulus, can necessitate reoperation. We present the case of a full-term newborn girl who underwent emergent Ladd’s procedure on Day 3 of life for malrotation with volvulus. She was discharged on postoperative Day 17 but returned the following day with recurrent bilious emesis. Imaging suggested partial obstruction. Exploratory laparotomy revealed recurrent volvulus with two twists at the duodenojejunal junction, which was successfully reduced. The patient recovered well and was discharged on postoperative Day 8. This case underscores the importance of clinical vigilance in detecting rare postoperative complications following Ladd’s procedure.

## Introduction

Malrotation is a congenital abnormality in intestinal rotation and fixation that occurs in ~1 in 500 live births [1]. Prompt diagnosis is crucial, as this can be life-threatening. The failure of the midgut to undergo normal 270° counterclockwise rotation can predispose to volvulus, a life-threatening complication [2]. The Ladd’s procedure, introduced in 1936, remains the standard of care for correcting malrotation [3]. Although generally successful, rare postoperative complications such as adhesions or recurrent volvulus can occur requiring prompt recognition and surgical intervention [4].

## Case report

A full-term newborn girl was delivered via elective cesarean section at 39 weeks’ gestation to a 28-year-old G2P1001 mother with full prenatal care. The pregnancy was notable for fetal findings of a dilated stomach and cavum septum pellucidum on ultrasound. Postnatally, the infant was diagnosed with patent foramen ovale and incontinentia pigmenti. She was born with a nuchal cord wrapped twice around her neck. APGAR scores were 7 and 9 at one and five minutes, respectively. The infant presented with a weak cry, cyanosis, and respiratory distress, requiring neonatal intensive care unit (NICU) admission and continuous positive airway pressure support.

**Figure 1:**
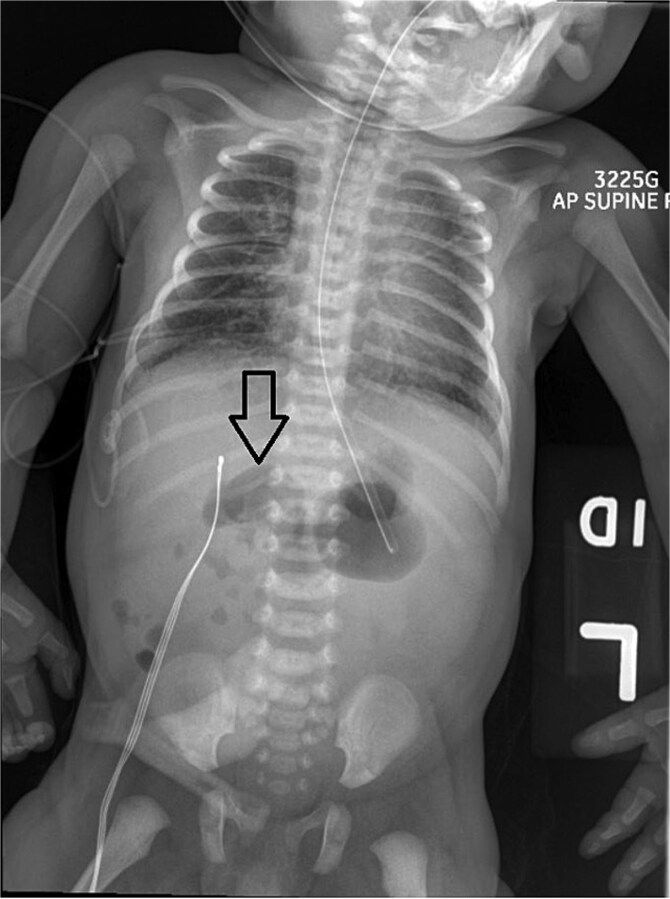
Abdominal X-ray shows nonspecific gas pattern with gas-filled, nondistended bowel loops on the right. Absence of small bowel gas on the left raises suspicion for malrotation.

**Figure 2:**
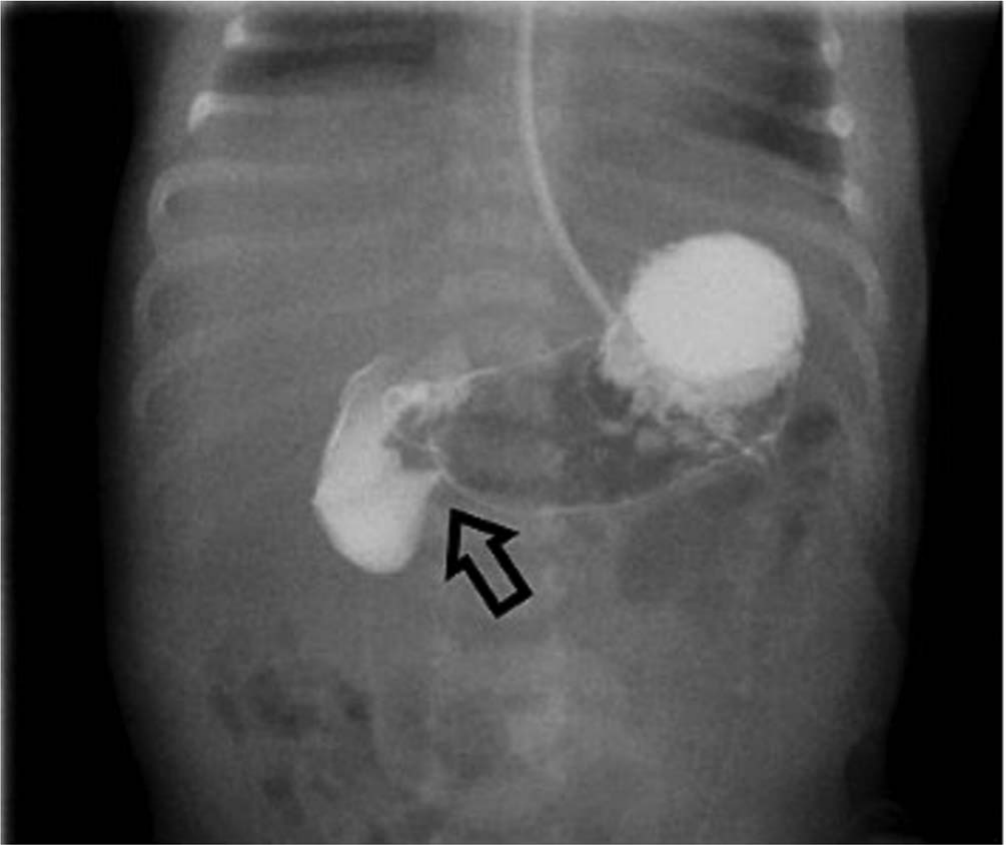
Initial UGI series demonstrates abnormally dilated descending duodenum with no contrast crossing the midline. Findings are consistent with obstruction.

On Day 3 of life, she developed multiple episodes of bilious emesis and passed meconium-stained stools. An abdominal radiograph revealed gas-filled, non-distended bowel loops on the right and absence of small bowel gas on the left (Fig. 1), raising suspicion for malrotation. An upper GI (UGI) series confirmed abnormal duodenal positioning and obstruction, suggestive of malrotation with volvulus (Fig. 2). Emergent Ladd’s procedure was performed, revealing midgut volvulus with cloudy peritoneal fluid but viable bowel.

The volvulus was reduced, Ladd’s bands were divided, and the cecum and colon were mobilized and repositioned to the left abdomen. A nasogastric tube confirmed no duodenal web. The infant remained in the NICU, was supported on total parenteral nutrition, and later transitioned to formula feeds. She was discharged on postoperative Day 17, tolerating feeds well.

**Figure 3:**
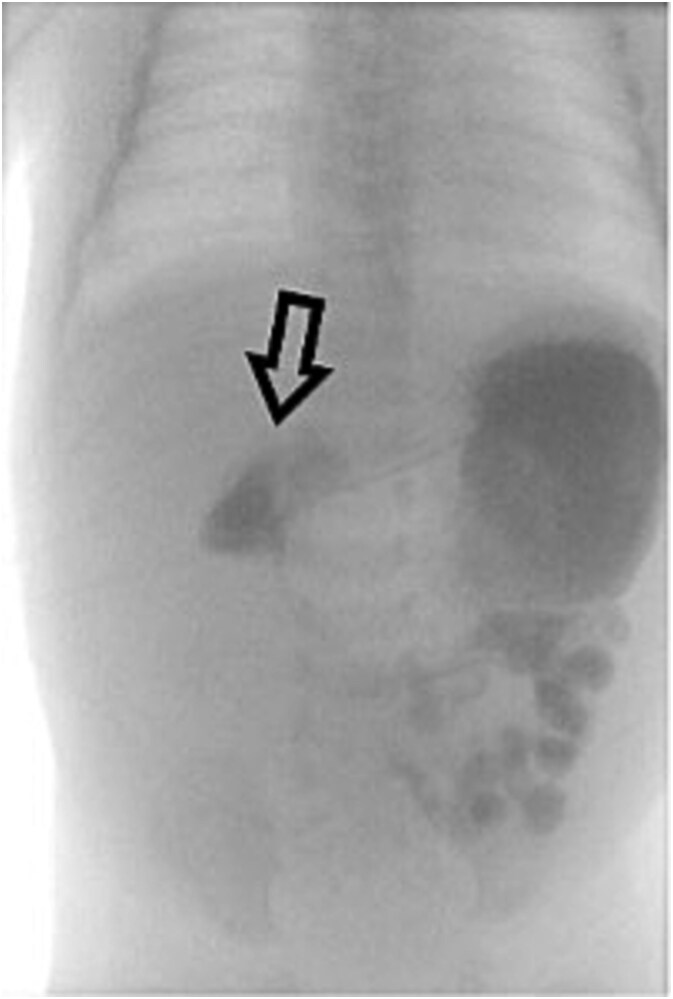
Repeat UGI series reveals delayed transit from the second to fourth portions of the duodenum, suggestive of partial obstruction likely due to adhesions.

However, she returned the next day with recurrent bilious vomiting. Abdominal X-ray demonstrated a paucity of bowel gas and a fluid-filled stomach. Repeat UGI series showed delayed transit from the second to fourth portions of the duodenum, suggesting a partial obstruction (Fig. 3). Exploratory laparotomy revealed recurrent midgut volvulus with two full twists at the duodenojejunal junction. The volvulus was successfully reduced, small adhesions lysed, and no bowel resection was required. The infant recovered uneventfully in the pediatric intensive care unit and was discharged on postoperative Day 8. She has been seen in outpatient clinic and is progressing well.

## Discussion

Congenital malrotation occurs in ~1 in 500 births, with volvulus developing in about 1 in 2500 cases [5]. Malrotation increases the risk of volvulus due to a narrowed mesenteric base, which may result in bowel ischemia, necrosis and death if untreated [5]. Clinical presentation in neonates often includes bilious emesis, feeding intolerance, or later, signs of peritonitis [6–8].

Diagnosis is typically made using abdominal radiographs and confirmed with UGI series [9, 10]. The Ladd’s procedure remains the surgical standard, repositioning the bowel and dividing Ladd’s bands. Appendectomy is often performed to avoid future diagnostic confusion, as the appendix is relocated [11].

Laparoscopic Ladd’s procedures offer benefits such as reduced postoperative pain, shorter recovery time, and fewer adhesions. However, some studies suggest a higher risk of volvulus recurrence compared to open approaches [12, 13]. A systematic review by Zhang *et al*. found no significant difference in recurrence between laparoscopic and open procedures, although surgeon experience and patient stability remain important considerations [13].

A recently introduced alternative to the Ladd’s procedure, known as Kareem’s procedure, offers a potentially lower risk of volvulus recurrence [14]. Kareem’s procedure involves placing the third and fourth parts of the duodenum behind the superior mesenteric axis to complete the 270° embryonic counterclockwise midgut rotation. It also includes fixation of the mesenteric attachments and, in selected patients with concomitant colonic dysmotility, possible colon resection [15]. However, widespread adoption of this technique has been limited due to its technical complexity and relative novelty [14]. The choice between an open or laparoscopic Ladd’s procedure—or even Kareem’s procedure—remains dependent on the individual surgeon.

Regardless of the approach, recurrent volvulus after a Ladd’s procedure is a rare but serious complication, occurring in 2%–7% of cases [15]. When volvulus recurs, emergency surgical intervention is required to detorse the bowel and prevent ischemia. One possible explanation for recurrence in our case is insufficient adhesion formation during the index operation. As described in the literature, development of adhesions during a Ladd’s procedure can help stabilize the repositioned bowel and reduce the risk of future volvulus or obstruction [15]. Strategies to promote adhesion formation intraoperatively include intentional abrasion of the parietal and visceral peritoneal surfaces, encouraging adherence of the realigned bowel [15]. This case highlights the importance of maintaining a high index of suspicion when postoperative symptoms such as bilious emesis reappear, as timely imaging and surgical management are critical to avoiding bowel compromise.

## Conclusion

Recurrent midgut volvulus after a Ladd’s procedure is rare but potentially life-threatening. This case highlights the importance of prompt recognition of postoperative complications and the necessity of immediate intervention. Continued clinical vigilance is essential, even in the early postoperative period.
